# Thermal and Mineral Sensitivity of Oil-in-Water Emulsions Stabilised using Lentil Proteins

**DOI:** 10.3390/foods9040453

**Published:** 2020-04-08

**Authors:** Loreto Alonso-Miravalles, Emanuele Zannini, Juergen Bez, Elke K. Arendt, James A. O’Mahony

**Affiliations:** 1 School of Food and Nutritional Sciences, University College Cork, T12 Y337 Cork, Ireland; 116221127@umail.ucc.ie (L.A.-M.); e.zannini@ucc.ie (E.Z.); e.arendt@ucc.ie (E.K.A.); 2 Fraunhofer Institute for Process Engineering and Packaging, Giggenhauser Str. 35, 85354 Freising, Germany; juergen.bez@ivv.fraunhofer.de

**Keywords:** lentil proteins, emulsion, mineral fortification, calcium, heat stability.

## Abstract

Oil-in-water emulsion systems formulated with plant proteins are of increasing interest to food researchers and industry due to benefits associated with cost-effectiveness, sustainability and animal well-being. The aim of this study was to understand how the stability of complex model emulsions formulated using lentil proteins are influenced by calcium fortification (0 to 10 mM CaCl_2_) and thermal processing (95 or 140 °C). A valve homogeniser, operating at first and second stage pressures of 15 and 3 MPa, was used to prepare emulsions. On heating at 140 °C, the heat coagulation time (pH 6.8) for the emulsions was successively reduced from 4.80 to 0.40 min with increasing CaCl_2_ concentration from 0 to 10 mM, respectively. Correspondingly, the sample with the highest CaCl_2_ addition level developed the highest viscosity during heating (95 °C × 30 s), reaching a final value of 163 mPa·s. This was attributed to calcium-mediated interactions of lentil proteins, as confirmed by the increase in the mean particle diameter (D[4,3]) to 36.5 µm for the sample with 6 mM CaCl_2_, compared to the unheated and heated control with D[4,3] values of 0.75 and 0.68 µm, respectively. This study demonstrated that the combination of calcium and heat promoted the aggregation of lentil proteins in concentrated emulsions.

## 1. Introduction

The world faces major challenges in food production and environmental sustainability over the next 30 years, with an expected growth of the world population to over nine billion people by 2050 [1,2,3]. The food system is responsible for more than a quarter of all greenhouse gas emissions and recent analyses have highlighted the environmental benefits of reducing the proportion of animal-derived food in our diets [4,5]. Furthermore, there is an increasing shift from animal-based to plant-based diets as the population becomes more conscious of the impact on ethical (e.g., animal welfare), health (e.g., antibiotics and hormones) and environmental (e.g., increase in carbon footprint) matters.

Legumes contain high amounts of protein, typically ranging between 20 and 40%, and are a rich source of essential amino acids such as leucine and lysine [6]. In particular, lentil seeds are showing promising results for the preparation of functional protein isolate ingredients, due to the absence of allergens, antinutritional compounds (e.g., isoflavones) and are also an affordable, sustainable and abundant raw material [7]. The major proteins present in lentils are globulins (~50%) and albumins (~16.8%), both considered globular proteins [8,9]. Globulins are constituted by vicilin-like, or trimeric (175–180 kDa), and legumin-like, or hexameric (300–370 kDa) proteins, having sedimentation coefficients of 7S and 11S, respectively. In contrast, the proteins in the albumin fraction have lower molecular weights (5–80 kDa) and are considered compact globular proteins [10].

Oil-in-water emulsion systems formulated with plant proteins are of particular interest to the food industry as the market for plant-based milks, yogurts, spreads, cheeses and infant formula is growing [11]. Recently, there has been a major need to identify surface-active plant protein sources that can be used in food formulations for reasons of sustainability and in satisfying dietary requirements [12]. While soy is the most established source of plant protein, proteins from several legumes, such as those from lentils, are growing in interest [13]. The proteins from pulses have been shown to contain amphiphilic proteins that form relatively thick interfacial layers around oil droplets, thereby enhancing emulsion formation and stability [14]. In this regard, lentil proteins were shown to be very effective natural emulsifiers for oil-in-water emulsion systems, being very stable to environmental and compositional stresses such as heat, pH and added salts [15]. These authors associated the high stability of lentil proteins with their surface hydrophobicity and/or formation of thick viscoelastic films around oil droplets, as also suggested by Can Karaca et al. (2015) [14]. In another recent study by Gumus et al. (2017b) [16] it was demonstrated that lentil proteins could be successfully applied to formulate oil-in-water emulsions, with such systems being used for bioactive lipid delivery, with similar behaviour to whey protein under simulated in vitro gastrointestinal digestion systems. Moreover, Avramenko et al. (2016) [17] produced lentil protein-based microcapsules using lentil proteins, with 4% protein (*w/w*), 36% maltodextrin (*w/w*) and 10% flaxseed oil (*w/w*).

Plant-based food products are often fortified with minerals, such as calcium, in order to meet the consumer needs and address their innately low calcium content [18]. Multivalent mineral ions (e.g., calcium), may precipitate from solution, promote precipitation of other components (e.g., proteins) or cause undesirable mouthfeel when used for fortification of liquid food systems [19,20,21]. In many such food applications, these mineral fortified systems are subjected to thermal processing (e.g., pasteurisation or sterilisation), during which proteins unfold, exposing reactive groups originally located in their interiors (e.g., nonpolar and sulfhydryl groups). Exposure of these reactive groups increases the attractive interactions between proteins that are adsorbed either on the same or on different oil droplet surfaces, causing droplet flocculation and coalescence in such emulsion-based systems. Stability and product quality challenges during processing and subsequent storage of nutritional beverages have been attributed to protein–protein and protein–mineral interactions, leading to age gelation and phase separation in dairy beverages [22,23].

To the authors knowledge, there is no information available on the influence of mineral fortification and thermal processing on reactivity and stability of lentil protein-stabilised emulsions, in particular in high solids (30%) systems; however, a large amount of information is available on similar dairy [24,25,26,27,28] and soy [29,30,31] protein-based emulsion systems. Therefore, the aim of this study was to determine the effect of calcium fortification (0–10 mM) and thermal processing (95 and 140 °C) on the reactivity, stability and quality of a model nutritional beverage emulsion formulated with a novel lentil protein ingredient.

## 2. Materials and Methods

### 2.1. Materials

Lentil protein isolate (85.1% protein; *w*/*w*), obtained by isoelectric precipitation, as described by Alonso-Miravalles et al. (2019) [7] was provided by the Fraunhofer Institute (Munich, Germany). Maltodextrin, with a dextrose equivalent (DE) value of 17 was supplied by Tereos (Lille, France). The sunflower oil was obtained from a local retail outlet (Tesco, Welwyn Garden City, Hertfordshire, United Kingdom). All the reagents used in this study were of analytical grade and supplied by Sigma-Aldrich (St. Louis, MO, USA), unless otherwise stated.

### 2.2. Preparation of Emulsions

Concentrated (target total solids ~30%) emulsions containing 4.75, 8.22 and 17.0 g/100 mL of lentil protein, sunflower oil and maltodextrin, respectively, were prepared as follows. The lentil protein was dispersed in preheated water (70 °C) using an overhead stirrer at 150 rpm for 1 h at 22 °C, after which the maltodextrin was added to the protein dispersion and mixed for 2 h under the same conditions. The mixture was adjusted to pH 6.8 and allowed to rehydrate at 5 °C for 18 h while mixing at 300 rpm by magnetic stirring. The temperature of the aqueous phase was then adjusted to 22 °C, pH measured, and readjusted to 6.8, if necessary. Sunflower oil was added to the aqueous phase to achieve a concentration of 8.22 g/100 mL and the mixtures were preheated to 50 °C before creating a coarse emulsion using an ultraturrax (T 25 Ultra-Turrax, Staufen, Germany) with a mixing speed of 12,000 rpm for 3 min. The coarse emulsion was then passed immediately through a homogeniser, twice, at 180 bar (1st and 2nd stage pressures of 150 and 30 bar, respectively). The emulsion was divided into seven different aliquots and 1 M CaCl_2_ was added, while magnetically stirring at 300 rpm, to give final calcium concentrations of 0, 2, 3, 4, 5, 6 and 10 mM. The pH of all aliquots was re-adjusted to pH 6.8 after adding CaCl_2_, checked, and readjusted, if necessary, after 1 h mixing at 22 °C. A standard pH meter (Meterlab, Radiometer Analytical, Villeurbanne, Lyon, France), with a PHM210 electrode, was used to measure pH at 22 °C after adding CaCl_2_.

### 2.3. Viscosity Changes on Heating

The viscosity changes during heat treatment of the different emulsions were determined using an AR-G2 controlled-stress rheometer equipped with a starch pasting cell geometry (TA Instruments Ltd., Water LLC, Leatherhead, Surrey, UK); the internal diameter of the cell was 36.0 mm, the diameter of the rotor was 32.4 mm, and the gap between the two elements at the geometry base was 0.55 mm. All measurements of viscosity were carried out at a fixed shear rate of 15 rad/s. The sample (28 g) was conditioned and held at 15 °C for 2 and 5 min, respectively, and the temperature increased to 95 °C (10 °C/min) and held at 95 °C for 30 s, after which the temperature was decreased to 15 °C (10 °C/ min) and maintained at this temperature for 5 min.

### 2.4. Heat Stability of the Emulsions

The heat stability at 140 °C of the emulsions was measured using the method of Davies and White (1966) [32] at different pH values in the range 6.3–7.2 (0.1 pH unit intervals) and different CaCl_2_ concentrations (i.e., 0 to 10 mM CaCl_2_). NaOH or HCl (1 M) was used for adjusting the pH. For the determination of heat stability, samples (2.5 mL) were placed in glass tubes (10 mm  ×  130 mm, AGB Scientific, Dublin, Ireland), sealed with silicone bungs, immersed in an oil bath thermostatically controlled at 140 °C (Elbanton B.V., Kerkdriel, the Netherlands), with continuous rocking at a motor speed setting of 3. The heat onset and coagulation time (HCT) was examined visually and taken as the time, in minutes, that elapsed between placing the sample in the oil bath and the formation of flecks or the complete coagulation of the sample, respectively.

### 2.5. Particle Size Distribution

The particle size distribution (PSD) of the different samples was measured before and after heating at 140 °C for 2 min. The PSD of the emulsions was measured using static laser light diffraction (Mastersizer 3000, Malvern Instruments Ltd., Worcestershire, UK). The refractive index was set at 1.47 and the absorption and dispersant refractive indices used were 0.001 and 1.33, respectively. The emulsions were equilibrated at 22 °C and introduced into the dispersing unit using ultrapure water as dispersant until a laser obscuration of 12% was achieved. The PSD was also measured using 0.2% sodium-dodecyl-sulphate as dispersant to differentiate between flocculation and coalescence.

### 2.6. Protein Profile Analysis

Protein profile was assessed using sodium dodecyl sulphate-polyacrylamide gel electrophoresis (SDS-PAGE) using precast gels (Mini-PROTEAN TGX, Bio-Rad Laboratories, Hercules, CA, USA) under nonreducing and reducing conditions, as follows. In order to understand which proteins preferentially remain in the serum phase of the emulsions (i.e., unadsorbed protein), the samples were centrifuged at 4000× *g* for 20 min and maintained at 5 °C for 2 h with the aim of solidifying the upper fat layer. The aqueous phase was collected carefully with a 1.2 × 40 mm needle (BD, Franklin Lakes, NJ, USA) and diluted to 4.5% (*v/v*) with ultrapure water. The separated aqueous phase was mixed (1:1; *v/v*) with the sample loading buffer which contained 65.8 mM Tris-HCl (pH 6.8), 26.3% (*w/v*) glycerol, 2.1% SDS and 0.01% bromophenol blue. The running buffer (10× Tris/Glycine/SDS, Bio-Rad Laboratories, Hercules, CA, USA) contained 25 mM Tris, 192 mM glycine and 0.1% SDS (*w/v*) at pH 8.3. The staining solution used was Coomassie Brilliant Blue R-250 (Bio-Rad Laboratories, Hercules, CA, USA). Sample solution (10 µL) was loaded into each well of the gel and run at a constant 150 V.

### 2.7. Confocal Laser Scanning Microscopy

Microstructural analysis of emulsions was performed using a Leica TCS SP Confocal Laser Scanning Microscope (Leica Microsystems, Heidelberg GmbH, Mannheim, Germany). Protein and lipid were fluorescently labelled with Nile Blue dye (Sigma-Aldrich, Dublin, Ireland). For the preparation of samples, 1 mL of the emulsion was mixed with 4 mL of low gelling temperature agarose (Sigma-Aldrich, St. Louis, MO, United States) solution (1.5%, *w/v*) at 30 °C, in order to prevent the movement of the oil globules during the analysis. Afterwards, 1 mL of the mixture was added to 50 µL of Nile Blue and incubated at 22 °C until the sample was solid. Visualisation of oil and protein in emulsions was carried out using an Ar laser (excitation 488 nm, emission 520–620 nm) and a He-Ne laser (excitation 633 nm, emission 650–730 nm) for oil (green) and protein (red), respectively. The observations were performed using 100× oil immersion objectives.

### 2.8. Statistical Data Analysis

All analyses were conducted in triplicate. The data generated was subject to one-way analysis of variance (ANOVA) using R i386 version 3.3.1 (R foundation for statistical computing, Vienna, Austria). A Tukey’s paired comparison test was used to determine statistically significant differences (*p* < 0.05) between mean values for different samples, at a 95% confidence level.

## 3. Results and Discussion

### 3.1. Influence of Calcium Chloride on pH of Emulsions

**Figure 1:**
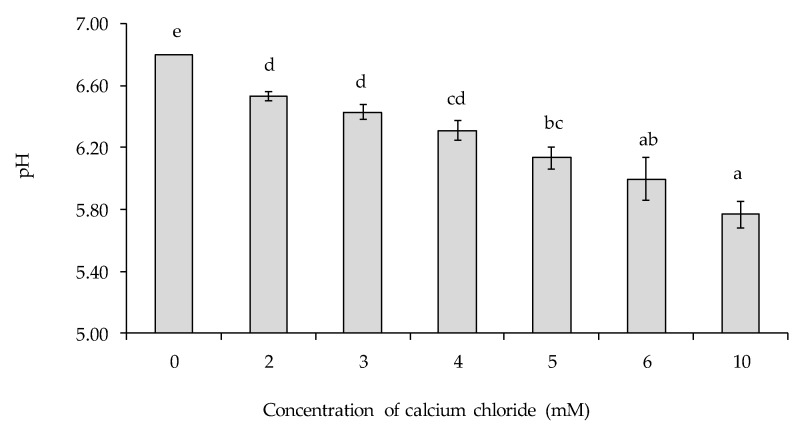
Influence of calcium chloride addition level, in the range 0–10 mM, on the pH of lentil protein-stabilised emulsions. ^(a–d)^ Samples not sharing a common letter differed significantly (*p* < 0.05).

Increasing addition level of CaCl_2_ resulted in a progressive decrease in pH of the emulsions (Figure 1). The decrease in pH with increasing CaCl_2_ concentration is likely due to a number of factors. Salts are known to shift the equilibrium constant of water and positively-charged salt ions may displace hydrogen ions from acidic groups on the proteins, which would result in a decrease in pH [25]. It should be noted that in all experiments in this study the pH of the emulsions was adjusted to 6.8 using 1 M HCl or NaOH before analysis.

### 3.2. Viscosity of Emulsions during Thermal Processing

**Figure 2:**
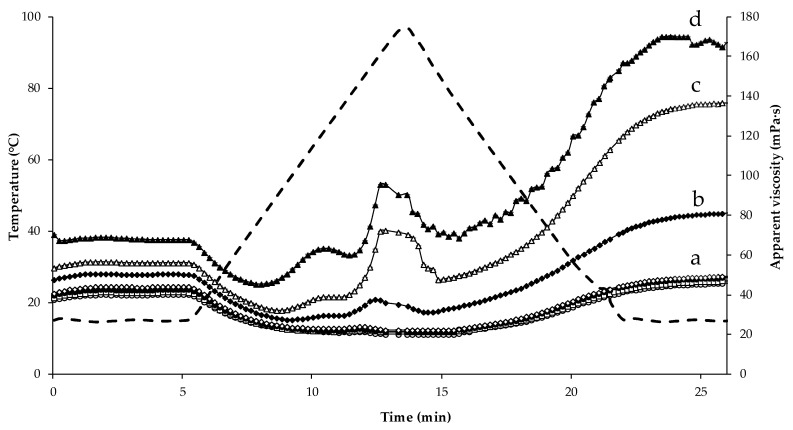
Apparent viscosity profiles of lentil protein-stabilised emulsions with 0 (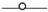), 2 (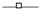), 3 (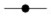), 4 (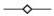), 5 (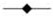), 6 (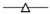) and 10 (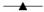) mM calcium chloride during heat treatment with peak temperature hold at 95 °C for 30 s using a starch pasting cell. Dashed line (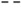) represents the temperature profile. ^(a–d)^ The statistical analysis was performed on the final viscosity of the samples; samples not sharing a common letter differed significantly (*p* < 0.05).

**Figure 3:**
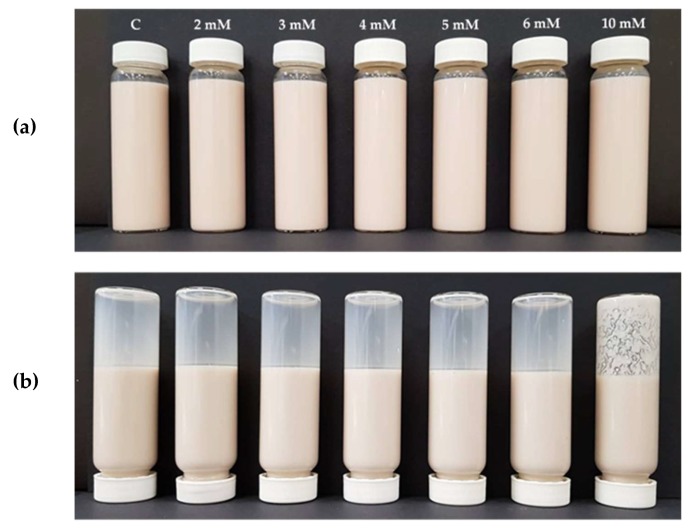
Photographs of lentil protein-stabilised emulsions containing. 0 (C), 2, 3, 4, 5, 6, 10 mM calcium chloride before (**a**) and after (**b**) heat treatment at 95 °C for 30 s.

The apparent viscosity of the lentil protein emulsions before heating (i.e., at 15 °C) increased with increasing addition level of CaCl_2_ with values of 37.8 and 70.4 mPa·s for CaCl_2_ concentrations of 0 and 10 mM, respectively (Figure 2). The emulsion stabilised by lentil proteins without CaCl_2_ addition presented a high stability to heat treatment. On increasing the temperature to 95 °C, the samples with 0–4 mM CaCl_2_ had lower viscosity in comparison with the samples containing 5–10 mM CaCl_2_. These samples (5, 6 and 10 mM CaCl_2_), had significantly (*p* < 0.05) higher viscosity (81.3, 136 and 163 mPa·s, respectively) after heat treatment compared to their initial viscosity. However, the samples with CaCl_2_ concentrations of 0–4 mM did not show significant differences (*p* < 0.05) between initial and final viscosity. The visual assessment of the emulsions after heating showed a destabilisation of the sample containing 10 mM CaCl_2_, with the presence of large flecks visible in the samples (Figure 3). On heating, a decrease in viscosity with increasing temperature is commonly observed for protein solutions; however, this normally continues until a protein-specific temperature is reached, at which point physical changes to the protein affect its structure (i.e., unfolding of polypeptide/peptide chain, disruption of hydrophobic interactions and aggregation by covalent and noncovalent bonding), generally causing an increase in viscosity [33] as seen in the samples with CaCl_2_ concentration in the range 5–10 mM. In this case, it was observed that the combination of heat and CaCl_2_ promoted changes in the emulsions, resulting in higher viscosity, especially in the emulsions with CaCl_2_ addition levels greater than 4 mM. Similar behaviour was observed for soya proteins in a study by Zhao et al. (2016) [29], where the authors observed that the combination of heat and CaCl_2_ promoted ionic interactions between the carboxyl groups of the amino acids mediated by calcium ions (Ca^2+^), facilitating formation of a gel network between soy proteins. In the case of dairy proteins, more specifically in whey protein solutions, Joyce et al. (2018) [28] observed an increase in the initial and final viscosity after heating at 85 °C when 2 mM CaCl_2_ was added, in comparison to the control sample without added calcium.

### 3.3. Heat Stability of Emulsions

**Figure 4:**
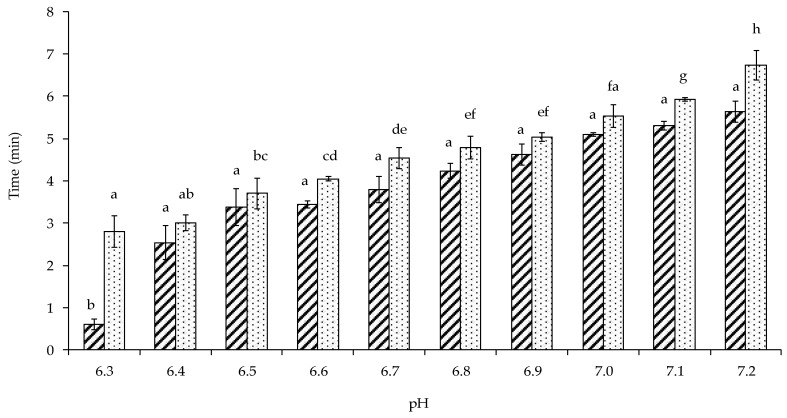
Influence of pH on the heat onset (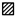) and coagulation (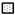) time at 140 °C, pH 6.8, of control (i.e., no calcium added) lentil protein-stabilised emulsions. ^(a–h)^ The statistical analysis was performed between heat onset or coagulation time results at different pH’s; samples not sharing a common superscript differed significantly (*p* < 0.05).

The heat stability of the control (0 mM added CaCl_2_) lentil protein-stabilised emulsion at different pH values was determined to gain a better understanding of its behaviour on heating at 140 °C. The heat coagulation time (HCT) increased from 2.8 to 6.7 min on increasing pH from 6.3 to 7.2 (Figure 4). Such heat-induced coagulation is governed by a balance between attractive and repulsive forces, as an increase in the latter has been shown to increase HCT [34]. Charge distribution among the amino acid side chains is altered by pH and in lentil proteins the negative charges increase with increasing pH, generating greater repulsion between lentil proteins [7]. Thus, at lower pH values, attractive forces will be stronger between the lentil proteins surrounding the oil globules, thereby reducing the HCT. Jeske et al. (2019) [35] reported a heat coagulation time of 17.4 min, under the same conditions used in this study, for a lentil protein-stabilised emulsion containing 3.3% and 3.3% fat and protein (*w/w*), respectively, after it was homogenised at 180 bar and heated at 65 °C for 30 min. The lower stability (i.e., <6 min) of the lentil protein-stabilised emulsion in the present study at pH 7.2 may be attributed to the higher total solids content (~30%).

**Figure 5:**
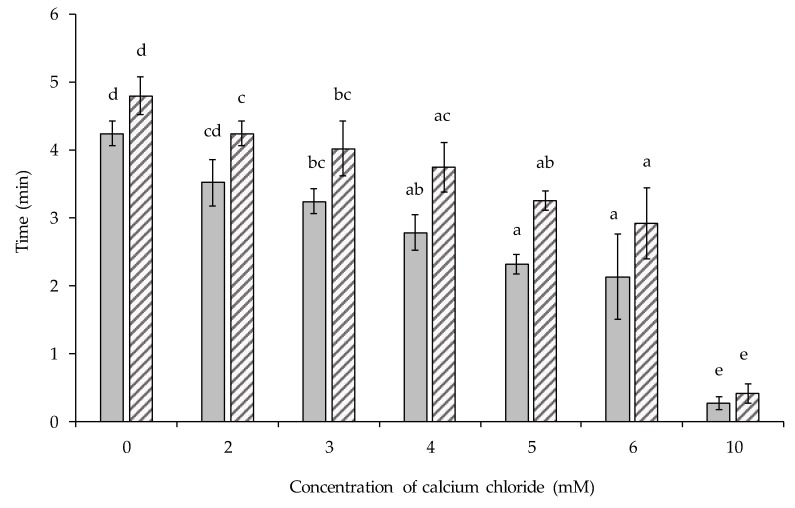
Influence of calcium chloride concentration on the heat onset (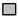) and coagulation (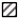) time of emulsions stabilised by lentil protein at pH 6.8. ^(a–e)^ The statistical analysis was performed between heat onset or coagulation time results at different [CaCl_2_]; samples not sharing a common superscript differed significantly (*p* ˂ 0.05).

The influence of CaCl_2_ addition level on the HCT of the lentil protein-stabilised emulsions was also evaluated in order to understand the effect of CaCl_2_ on the HCT at 140 °C (Figure 5). From this data, it could be observed that, as the concentration of added CaCl_2_ increased, the HCT decreased from 4.8 min (no CaCl_2_ added) to 2.9 min (6 mM CaCl_2_). Further increases of CaCl_2_ to 10 mM resulted in an almost instantaneous coagulation of the emulsions after insertion in the oil bath. The behaviour displayed by the lentil protein-stabilised emulsions has also been observed in bovine milk where HCT is inversely related to the concentrations of divalent cations, such as calcium and magnesium [24]. Omoarukhe et al. (2010) [27] investigated the effects of different calcium salts on the heat stability of bovine milk and observed that heat stability was reduced on adding CaCl_2_. In relation to plant proteins, several authors [29,31] have studied the effect of CaSO_4_ on the formation of soy protein networks, reporting that CaCl_2_ increased gel strength by the formation of ionic bridges between soy proteins. Furthermore, in soy milk, coagulation of the proteins was observed when 25 mM CaCl_2_ was added [30]. In the same way, in a recent study by Silva et al. (2019) [21], the calcium-binding capacity of soy and pea proteins was demonstrated, concluding that both proteins were able to bind more calcium than whey proteins, contributing to an increase in critical gelation temperature of micellar caseins in mixed plant-milk protein systems.

### 3.4. Particle Size Distribution of Emulsions

**Figure 6:**
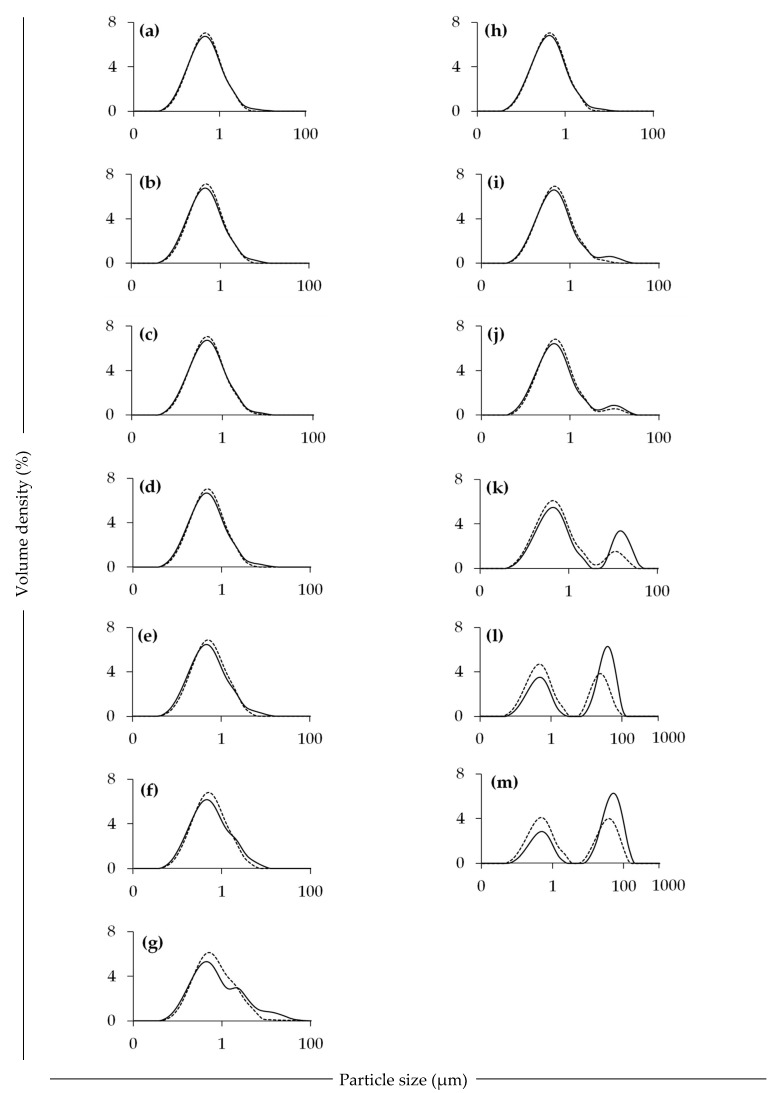
Particle size distribution of lentil protein-stabilised emulsions before (a-g) and after (h-m) heat treatment at 140 °C for 2 min, pH 6.8, without (solid line) or with (dashed line) sodium dodecyl sulphate. Samples had 0 (a and h), 2 (b and i), 3 (c and j), 4 (d and k), 5 (e and l), 6 (f and m) and 10 (g) mM added calcium chloride.

The particle size distribution of the control emulsion without added CaCl_2_, and with different CaCl_2_ addition levels was measured before and after heating at 140 °C for 2 min (Figure 6). The unheated emulsion with no added CaCl_2_ showed a monomodal droplet size distribution, with a volume-weighted mean particle diameter (D[4,3]) value of 0.75 µm. There was no significant difference in the D[4,3] value (0.75–0.93 µm) between the 0 and 6 mM CaCl_2_ containing samples; however, the D[4,3] value (2.30 µm) was significantly higher in the sample with 10 mM added CaCl_2_. Keowmaneechai and McClements (2002) [25] obtained a mean particle diameter of 0.70 µm for whey protein-stabilised oil-in-water emulsions and observed an increase in PSD with the addition of CaCl_2_, in particular on increasing CaCl_2_ concentration ≥4 mM. The authors associated the increase in particle size to flocculation, rather than coalescence, as the ions reduce the electrostatic repulsion between oil droplets, enabling the droplets to associate. Other authors have shown that the addition of CaCl_2_ to soy proteins increases their particle size [36] and facilitates the formation of cold-set protein gels [37].

After heating the emulsions at 140 °C for 2 min, with no added CaCl_2_, the PSD remained stable. Gumus et al. (2017a) [15], reported that lentil protein-stabilised emulsions are stable to aggregation across a temperature range of 20 to 90 °C, and related this to their hydrophobicity or thickness of the interfacial protein layer. The results reported earlier (Section 3.2) are in agreement with this, where no increase in viscosity was observed at 95 °C in the sample with 0 mM CaCl_2_. However, when CaCl_2_ and heat were combined, the appearance of a second population of larger particles (10–100 µm) was observed, especially at CaCl_2_ concentrations ≥4 mM (Figure 6), suggesting association of the primary emulsion droplets. The Dv(90) (particle size below which 90% of sample volume is found) of the samples, both before and after heating at 140 °C for 2 min, increased progressively with increasing CaCl_2_ addition levels, indicating the formation of larger particles in the samples.

Furthermore, in order to understand the nature of the interactions between the droplets within the heat-treated emulsions, the PSD was also measured using 0.20% sodium-dodecyl-sulphate (SDS) as dispersant. In the presence of SDS, the mean particle size of the droplets could be reduced significantly, implying that the original increase in particle size was, at least partially, due to flocculation (i.e., reversible upon the use of dissociating agent), and not coalescence, of oil droplets in the emulsions [23]. 

### 3.5. Protein Profile Analysis

**Figure 7:**
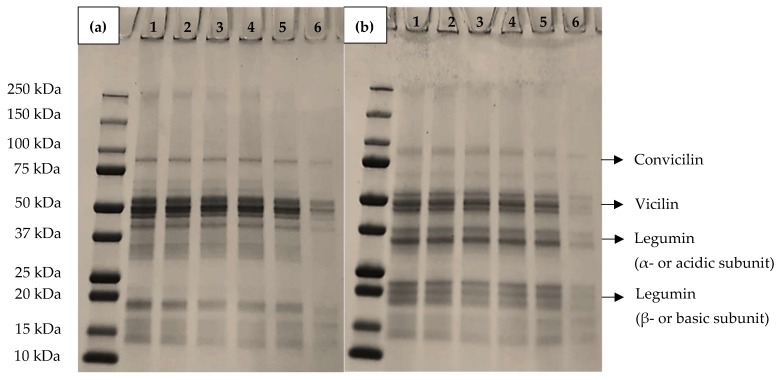
Representative sodium dodecyl sulphate-polyacrylamide gel electrophoretograms (SDS-PAGE) of the serum phase after centrifugation of lentil protein-stabilised emulsions with calcium chloride concentrations of 0 mM (1), 2 mM (2), 3 mM (3), 4 mM (4), 5 mM (5) and 6 mM (6) under nonreducing (**a**) and reducing (**b**) conditions. The sample with 10 mM calcium chloride added was not included in the Figure as no bands were detected in the gel. The first lane of the gel contains the molecular weight marker (MW).

Sodium dodecyl sulfate–polyacrylamide gel electrophoresis analysis under nonreducing and reducing conditions after centrifugation of the emulsions with different addition levels of CaCl_2_ are shown in Figure 7. The total quantity of protein in the serum phase, after centrifugation, reduced with increasing CaCl_2_ addition and this was most notable in the sample with 6 mM CaCl_2_. These results suggest that the lentil proteins are interacting with each other, and possibly the interfacial layer proteins, especially with increasing CaCl_2_ addition level, causing their migration with the oil droplets in the cream phase, as no sediment was notable in the samples on centrifugation. The protein profile was compared to that performed for the raw material [7], observing no differences in the different molecular weight bands. In the samples with CaCl_2_ concentrations between 0 and 5 mM, proteins with molecular weight (MW) of ~50, ~37 and ~20 kDa under nonreducing conditions were observed. The bands with MW ~50 kDa may correspond to vicilin subunits, which is a 7S trimeric protein with a MW of 150 kDa, one of the major globulins, together with legumin, found in many pulses. Each trimer of vicilin has a MW of 50 kDa without disulfide bridging [38]. The bands at 37 and 25 kDa correspond to the acidic and basic subunits of legumin, in accordance with previous studies [39,40]. Legumin, an 11S globulin, is a hexameric protein formed by subunits with MW ~60 kDa, which consist of acidic (~40 kDa) and basic (~20 kDa) subunits, linked by disulfide bonds [41,42]. Under reducing conditions, similar profiles were observed, although bands at 37 and 25 kDa were slightly more intense, with the disappearance of some high MW bands at ~50 kDa. This can be associated with the dissociation of legumin into its acidic (MW ~40 kDa) and basic (~20 kDa) subunits by the reduction of disulfide bonds in the presence of β-mercaptoethanol. This result suggests that all of the proteins are involved in the same proportions in emulsion formation; however, the reduction in intensity of the bands indicates that the proteins are interacting with the CaCl_2_, being displaced from the serum phase.

### 3.6. Confocal Laser Scanning Microscopy

**Figure 8:**
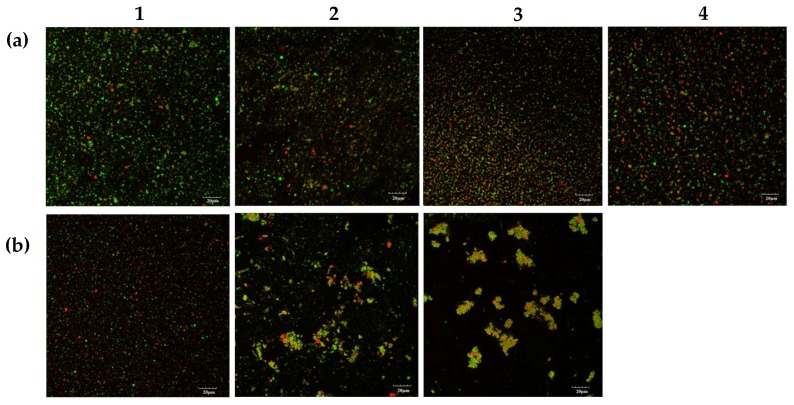
Confocal laser scanning micrographs of emulsions (**a**) before and (**b**) after heat treatment in an oil bath at 140 °C for 2 min with 0 (a_1_ or b_1_), 4 (a_2_ or b_2_), 6 (a_3_ or b_3_) and 10 (a_4_) mM calcium chloride. The 10 mM sample after heating was not analysed due to extensive aggregation. Protein = red; oil = green. Scale bar (bottom right) is 20 µm.

Selected micrographs, as obtained by confocal laser scanning microscopy (CLSM), are displayed in Figure 8. CLSM showed that the emulsion without added CaCl_2_ and heat treatment had fine and uniformly distributed oil droplets. Jeske et al. (2019) [35] also observed homogenously distributed oil droplets in pasteurised lentil protein-stabilised emulsions homogenised at 180 bar. Development of a small number of larger oil droplets was observed as the concentration of CaCl_2_ increased to 10 mM in the unheated calcium-fortified emulsion samples. Interactions could be observed between the proteins, with formation of protein aggregates entrapping the oil globules. This suggests that CaCl_2_ promotes interactions between lentil proteins, causing aggregation and proximity between the oil globules. These results are in agreement with PSD analysis of the oil droplets where the mean particle size increased considerably with 10 mM CaCl_2_ addition. Ye and Singh (2000) [26] observed similar behaviour in 0.5% whey protein-stabilised emulsions with concentrations of 3 mM CaCl_2_ and attributed this to protein-mediated bridging flocculation.

With the CLSM of the emulsions after being heated for 2 min, the formation of protein aggregates, and an increase in oil globule size, was evident at CaCl_2_ addition levels ≥4 mM (Figure 8b_2_). In particular, the formation of a dense protein network structure entrapping flocculated oil droplets could clearly be seen in the heated 6 mM CaCl_2_ sample (Figure 8b_3_). These observations are in agreement with the results obtained for particle size and viscosity analysis , where an increase in PSD and viscosity were observed, on heating at CaCl_2_ addition levels ≥4 mM. The CLSM analysis confirmed that the increase in particle size was mostly due to flocculation, but also to coalescence, as a small number of larger oil droplets were observed at higher CaCl_2_ addition levels.

## 4. Conclusions

The influence of calcium fortification and thermal processing of a concentrated lentil protein-stabilised oil-in-water emulsion was studied. The sample without calcium chloride addition was very stable at heat treatments of 95 and 140 °C. However, the addition of calcium, in particular at concentrations greater than 4 mM, led to reduced heat stability of the emulsions, as demonstrated by increases in particle size and viscosity. Confocal laser scanning microscopy confirmed that the increase in particle size and viscosity were due largely to flocculation of oil globules. These results can be applied in the development of novel plant-based food products such as infant, clinical and elderly nutritional products.
